# Particulate Air Pollution, Oxidative Stress Genes, and Heart Rate Variability in an Elderly Cohort

**DOI:** 10.1289/ehp.10318

**Published:** 2007-08-20

**Authors:** Teresa Chahine, Andrea Baccarelli, Augusto Litonjua, Robert O. Wright, Helen Suh, Diane R. Gold, David Sparrow, Pantel Vokonas, Joel Schwartz

**Affiliations:** 1 Department of Environmental Health, Harvard School of Public Health, Boston, Massachusetts, USA; 2 Center of Molecular Epidemiology and Genetics; and EPOCA Epidemiology Research Center, University of Milan and IRCCS Maggiore Hospital, Mangiagalli and Regina Elena Foundation, Milan, Italy; 3 Channing Laboratory, Department of Medicine, Brigham and Women’s Hospital, Harvard Medical School, Boston, Massachusetts, USA; 4 VA Normative Aging Study, Veterans Affairs Boston Healthcare System and the Department of Medicine, Boston University School of Medicine, Boston, Massachusetts, USA

**Keywords:** air particles, air pollution, cardiovascular health, genetic variation, *GST*, heart rate variability, *HMOX-1*, PM_2.5_

## Abstract

**Background and objectives:**

We have previously shown that reduced defenses against oxidative stress due to glutathione *S*-transferase M1 (*GSTM1*) deletion modify the effects of PM_2.5_ (fine-particulate air pollution of < 2.5 μm in aerodynamic diameter) on heart rate variability (HRV) in a cross-sectional analysis of the Normative Aging Study, an elderly cohort. We have extended this to include a longitudinal analysis with more subjects and examination of the GT short tandem repeat polymorphism in the heme oxygenase-1 (*HMOX-1*) promoter.

**Methods:**

HRV measurements were taken on 539 subjects. Linear mixed effects models were fit for the logarithm of HRV metrics—including standard deviation of normal-to-normal intervals (SDNN), high frequency (HF), and low frequency (LF)—and PM_2.5_ concentrations in the 48 hr preceding HRV measurement, controlling for confounders and a random subject effect.

**Results:**

PM_2.5_ was significantly associated with SDNN (*p* = 0.04) and HF (*p* = 0.03) in all subjects. There was no association in subjects with *GSTM1*, whereas there was a significant association with SDNN, HF, and LF in subjects with the deletion. Similarly, there was no association with any HRV measure in subjects with the short repeat variant of *HMOX*-1, and significant associations in subjects with any long repeat. We found a significant three-way interaction of PM_2.5_ with *GSTM1* and *HMOX-1* determining SDNN (*p* = 0.008), HF (*p* = 0.01) and LF (*p* = 0.04). In subjects with the *GSTM1* deletion and the *HMOX-1* long repeat, SDNN decreased by 13% [95% confidence interval (CI), −21% to −4%], HF decreased by 28% (95% CI, −43% to −9%), and LF decreased by 20% (95% CI, −35% to −3%) per 10 μg/m^3^ increase in PM.

**Conclusions:**

Oxidative stress is an important pathway for the autonomic effects of particles.

Particulate air pollution (PM) is associated with increased risk of hospitalization and death from cardiovascular disease (Brook et al. 2004; Forastiere et al. 2005; Samet et al. 2000; Schwartz 1999; Zanobetti and Schwartz 2005), but the mechanisms underlying such effects are not fully understood. Reductions in heart rate variability (HRV), a noninvasive measure that independently predicts cardiovascular mortality (Tsuji et al. 1996), have been related to PM exposure, particularly to fine-particulate air pollution of < 2.5 μm in aerodynamic diameter (PM_2.5_) (Creason et al. 2001; Devlin et al. 2003; Gold et al. 2000; Holguin et al. 2003; Liao et al. 2005a; Magari et al. 2002; Park et al. 2005; Pope et al. 2004; Schwartz et al. 2005a).

Animal experiments indicate that reactive oxygen species (ROS), which have established relevance in the pathogenesis of cardiovascular disease (Dhalla et al. 2000), are potential mediators for particle effects on HRV and other cardiovascular end points (Brook et al. 2004; Gurgueira et al. 2002; Nel 2005; Rhoden et al. 2004). While animal models can identify potential mechanisms of particle effects, the relative importance of these pathways in humans at lower doses is not clear and may be determined by examining subjects with genetically determined differences in oxidative-stress defenses. In elderly subjects living in the Boston, Massachusetts, metropolitan area, we recently showed that PM_2.5_ levels during the 48 hr before the study were associated with decreased HRV in individuals with the glutathione *S*-transferase M1 (*GSTM1*) deletion, but had no effect in subjects with *GSTM1* present (Schwartz et al. 2005b).

Studies showing the effect of just one polymorphism are unlikely to correctly represent the complex etiology of common diseases, and failure to account for gene–gene interactions in the search for susceptibility genes has been widely suggested to explain the persisting difficulties in replicating significant findings (Millstein et al. 2006). Particle exposure induces both heme oxygenase-1 (*HMOX-1)* and *GSTM1* expression through activation of the genetic antioxidant response element (ARE) (Li et al. 2004). A high number of microsatellite (GT)n dinucleotide repeats in 5′-flanking region may reduce *HMOX-1* inducibility by ROS and has been associated with increased risk of coronary artery disease in high-risk groups with hyperlipidemia, diabetes, or current smoking (Chen et al. 2002; Kaneda et al. 2002). Consequently, individuals with a high number of (GT)n repeats may be more susceptible to the effects of airborne particles.

We hypothesized that the gene encoding HMOX-1, which is involved in various aspects of responses against oxidative stress, may *a*) directly modify the effect of ambient PM on HRV and *b*) interact with *GSTM1* to determine which subjects are susceptible to airborne particle effects. To establish the role of the antioxidant response pathway in determining the cardiovascular effects of airborne particles, we examined in the present study the association of PM_2.5_ with HRV in a repeated measure study of elderly subjects from the Boston metropolitan area, and evaluated how that association was affected by genetic variation in the *HMOX-1* and *GSTM1* loci.

## Materials and Methods

### Study population

Our study population consisted of 539 white males from the Normative Aging Study (NAS), a longitudinal study of aging established in 1963 by the U.S. Veterans Administration (Bell et al. 1972). Between January 2000 and June 2005, all participants still presenting for examination (*n* = 676) were evaluated for HRV. Of these, 137 subjects were excluded because of heart arrhythmias, measurement time < 3.5 min, or missing potential confounding variables or *HMOX-1* data. Among the remaining 539 subjects, *GSTM1* data were available for 476 subjects, who had one (*n* = 314) or two (*n* = 162) HRV measurements. In subjects with multiple HRV measurements, the time interval between measurements was approximately 3 years. This study was conducted in compliance with all applicable requirements of the U.S. and international regulations (including institutional review board approval). All subjects gave written informed consent prior to the study.

### HRV measurement

HRV was measured at rest during normal breathing for 7 min using a two-channel (five-lead) ECG monitor (Trillium 3000; Forest Medical, East Syracuse, NY) while the subject was seated. Standard deviation of normal-to-normal intervals (SDNN), high frequency (HF) (0.15–0.4 Hz), and low frequency (LF) (0.04 –0.15 Hz) were computed with a fast Fourier transform using software (Trillium 3000 PC Companion Software; Forest Medical) complying with established guidelines (Task Force of the European Society of Cardiology and the North American Society of Pacing and Electro-physiology 1996). In the analysis, we used the 4 consecutive minutes of ECG reading that included the lowest number of artifacts.

### Air pollution and weather data

Continuous PM_2.5_ was measured at a stationary monitoring site on the roof of Countway Library of Harvard University in downtown Boston using a Tapered Element Oscillating Microbalance (TEOM; Model 1400A, Rupprecht & Patashnick Co., East Greenbush, NY). Meteorologic data was obtained from the Boston airport weather station. The 48-hr moving average of PM_2.5_ before each HRV measurement was used as the exposure index, as this exposure period has shown the strongest association in previous studies (Park et al. 2005).

### HMOX-1 *and* GSTM1 *genotyping.*

The *GSTM1* locus (UniGene Hs.301961; UniGene 2007a) was amplified at exons 4 and 5 by polymerase chain reaction (PCR) as previously described to differentiate between the null polymorphism and the presence of one or more copies of the gene (Schwartz et al. 2005b). The *HMOX-1* (UniGene Hs.517581; UniGene 2007b) microsatellite (GT)n length assay was designed per Yamada and coworkers (Yamada et al. 2000). Briefly, the *HMOX-1* locus was amplified by PCR at the 5′ promoter flanking region containing (GT)n repeats with primers as described by Yamada, and the sizes of the PCR products were analyzed with a laser-based automated DNA sequencer (AB 3100; Applied Biosystems, Foster City, CA). Although the exact cutoff for *HMOX-1* modulation is still unknown, constructs with lengths of > 25 repeats showed reduced *HMOX-1* basal promoter activity and decreased transcriptional upregulation in response to various stimuli like H_2_O_2_ compared with lengths < 25 repeats (Chen et al. 2002; Yamada et al. 2000). In the analysis of the data, we used the 25-repeat cutoff to categorize the study subjects in two categories [< 25 (GT)n repeats in both alleles or ≥ 25 (GT)n repeats in at least one allele] based on the *HMOX-1* microsatellite length.

### Statistical analysis

HRV measurements were log_10_-transformed to improve normality. The following potential confounders were chosen *a priori* and included in the analysis: age, body mass index (BMI), mean arterial pressure, fasting blood glucose, cigarette smoking (never/former/current), alcohol consumption (≥ 2 drinks a day, yes/no), use of beta-blockers, calcium channel blockers, acetylcholinesterase (ACE) inhibitors, room temperature, season, and 48-hr moving average of outdoor apparent temperature. Potential nonlinearity between apparent temperature and HRV was accounted for using a linear and quadratic term.

Because our data included repeated measures of HRV for many participants, our data may lack independence. To deal with this, we fit a mixed effects model (PROC MIXED in SAS version 9.0; SAS Institute Inc., Cary, NC). We assumed:


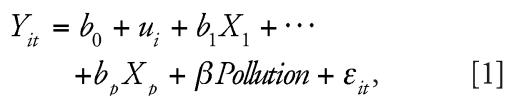


where *Y_it_* is the logarithm of HRV in subject *i* at time *t*, *b*_0_ is the overall intercept, and *u_i_* is the separate random intercept for subject *i.* In the above, *X*_1_*—X_p_* are the covariates measured at each of the visits in which the HRV measurements were taken. This captures the correlation among measurements within the same subject.

## Results

Table 1 shows the levels and distribution of the variables used in this study, overall, and for the different combinations of *GSTM1* genotype and *HMOX 1* microsatellite repeat length. The study participants were all male, with average age of 72.8 years (SD = 6.6 years) at the first HRV measurement. No differences among the subpopulations defined by the combinations of *GSTM1* genotype [wild-type or null] and *HMOX-1* microsatellite repeat length [< 25 (GT)n repeats in both alleles or ≥ 25 (GT)n repeats in at least one allele] were found in age, BMI, systolic blood pressure, diastolic blood pressure, mean arterial pressure, heart rate, fasting blood glucose, total cholesterol, high-density lipoproteins (HDL), triglyceride, smoking status, alcohol intake (≥ 2 drinks/day), history of coronary heart disease (CHD), diabetes, hypertension, or stroke, and use of beta-blockers, calcium channel blockers, or ACE inhibitors (Table 1). The two genes were not associated with each other (*p* > 0.67).

Table 2 shows the results of the analyses for the association of PM_2.5_ with changes inHRV for the entire population (model 1), by *GSTM1* genotype (model 2), and by *HMOX-1* microsatellite (GT)n repeat length (model 3).

For the entire population, we found that a 10 μg/m^3^ increase in ambient PM_2.5_ in the 48 hr before the HRV measurement was associated with a 6.8% decrease in SDNN [95% confidence interval (CI), −12.9 to −0.2; *p* = 0.043] and with a 17.3% decrease in HF (95% CI, −30.0 to −2.3; *p* = 0.026). Ambient PM_2.5_ concentrations were also negatively associated with LF (estimated change = −11.2%, 95% CI, −22.8 to 2.2), but the result was not statistically significant (*p* = 0.10).

The PM_2.5_–HRV association was modified by *GSTM1* genotype, with PM_2.5_ concentrations negatively associated with SDNN, HF, and LF in *GSTM1*-null subjects, whereas no association between PM_2.5_ and HRV was found in *GSTM1*–wild-type carriers. In subjects with the *GSTM1*-null deletion, a 10-μg/m^3^ increase in PM_2.5_ was associated with a 10.5% decrease in SDNN (95% CI, −18.2 to −2.2; *p* = 0.015), a 24.2% decrease in HF (95% CI, −39.2 to −5.5; *p* = 0.014), and a 17.0% decrease in LF (95% CI, −31.0 to −0.2; *p* = 0.048). In *GSTM1*–wild-type subjects, the estimated decreases in HRV for a 10-μg/m^3^ increase in PM_2.5_ were 2.0% (95% CI, −11.3 to 8.3; *p* = 0.69) for SDNN, 4.0% (95% CI, −24.8 to 22.6; *p* = 0.74) for HF, and 0.6% (95% CI, −19.0 to 22.0; *p* = 0.95) for LF. However, the *p*-values for statistical interactions between PM_2.5_ and *GSTM1* genotype were not significant.

Similarly, we found that that PM_2.5_–HRV association was modified by *HMOX-1* genotypes. Ambient PM_2.5_ concentrations were negatively associated with all three HRV outcomes in carriers of at least one allele with ≥ 25 microsatellite (GT)n repeats in the *HMOX-1* promoter region, whereas no association between PM_2.5_ and HRV was present in carriers of < 25 repeats in both alleles. In subjects with at least one allele with ≥ 25 microsatellite (GT)n repeats, a 10-μg/m^3^ increase in PM_2.5_ was associated with a 8.5% decrease in SDNN (95% CI, −14.8 to −1.8; *p* = 0.014), a 20.1% decrease in HF (95% CI, −32.9 to −5.0; *p* = 0.012), and a 14.0% decrease in LF (95% CI, −25.7 to −0.5; *p* = 0.043). The *p*-value for statistical interactions between PM_2.5_ and *GSTM1* genotype was marginally significant (*p* = 0.059) when the SDNN component of HRV was considered but was not significant for HF (*p* = 0.14) and LF (*p* = 0.11).

We further evaluated the interrelationship between PM_2.5,_ *GSTM1*, and *HMOX-1* by estimating the effect of PM_2.5_ on HRV within each combination of the *GSTM1* genotypes and *HMOX-1* microsatellite repeat length categories (Table 3). These results indicate a clear trend of increasingly negative coefficients as we move across gene categories. In carriers of both the *GSTM1*-null deletion and at least one allele with ≥ 25 *HMOX-1* microsatellite (GT)n repeats, PM_2.5_ was negatively associated with all three HRV outcomes, whereas no significant association was found in subjects with any other combinations. In subjects carrying the *GSTM1*-null deletion and at least one allele with ≥ 25 *HMOX-1* microsatellite (GT)n repeats, a 10-μg/m^3^ increase in PM_2.5_ in the 48 hr before the HRV measurement was associated with a −12.7% decrease in SDNN (95% CI, −20.6 to −3.9; *p* = 0.0059), a 27.8% decrease in HF (95% CI, −43.0 to −8.5; *p* = 0.0073), and a 20.1% decrease in LF (95% CI, −34.5 to −2.7; *p* = 0.0261). *GSTM1* genotypes and *HMOX-1* microsatellite repeat lengths had a combined effect on the association between PM_2.5_ and HRV, as shown in Table 3 by the significant three-way interaction term between *GSTM1*, *HMOX-1*, and PM_2.5_ (coded as PM_2.5_ × a trend variable that is 1 for *GSTM1* present and both short repeats, 2 for *GSTM1* null and both short repeats, 2 for one long repeat and *GSTM1* present, and 4 for *GSTM1* null and at least one long repeat).

**Figure 1:**
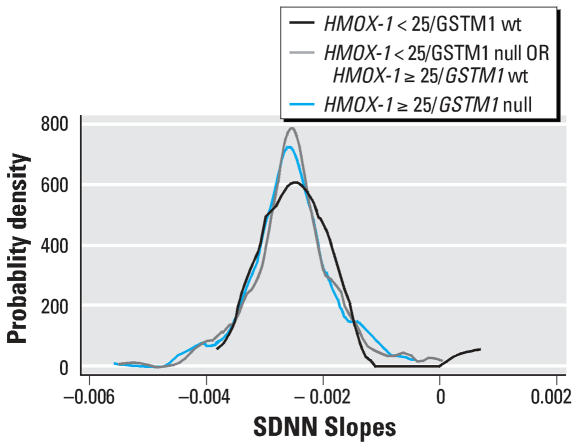
Kernel density plot of subject-specific random slopes for the association of PM_2.5_ level with SDNN, by *GSTM1* genotype and *HMOX-1* repeat length.

To test whether the effect modification by genotype was driven by a few individuals or represented a more general shifting of the distribution of PM_2.5_–HRV slopes, we refit our mixed models, dropping the interaction terms with genotype but allowing for random subject-specific slopes. Figure 1 shows the distribution of subject-specific PM_2.5_ slopes for SDNN by three categories of genotype (*HMOX-1* < 25 repeats and *GSTM1* wt, either *HMOX-1* ≥ 25 repeats or *GSTM1* null, or both *HMOX-1* ≥ 25 repeats and *GSTM1* null). The shift to the left of the distributions does not appear to be driven by outliers. The same shift in the distributions was observed also for the HF and LF components (data not shown).

Goodness of fit of the three-way interaction models was evaluated using the Akaike Information Criterion (AIC). AICs for models including the three-way interaction term were lower than those obtained from corresponding models including the main effects of GSTM1, HMOX-1, and PM_2.5,_ as well as the two-way interactions between *GSTM1* and PM_2.5_ and *HMOX-1* and PM_2.5,_ thus indicating better goodness of fit.

## Discussion

Our study, based on an elderly population in Boston, showed that functional genetic variations in *GSTM1* and *HMOX-1*, both of which are related to defenses against oxidative stress, modify the effects of PM_2.5_ on HRV. In the present work, we have extended our previous results examining the modification of the PM_2.5_–HRV association by *GSTM1* (Schwartz et al. 2005b) to include other HRV outcomes and repeated measures on subjects, to show effect modification by *HMOX-1*, and to show a three-way interaction between the two genes and combustion particles.

This work is part of a series of studies seeking to examine the potential pathways by which particles affect HRV. Specifically, we are looking at oxidative stress and endothelial function as potential pathways to this outcome. We hypothesize that if a pathway is important in the effect of PM_2.5_ on HRV, then factors that modify that pathway, either genes or drugs, may modify the PM_2.5_ response. We are also looking at metal-processing pathways as an indirect test of the hypothesis that metals on the PM_2.5_ particles play an important role in the HRV response (Park et al. 2006).

In our previous work on the same population we showed that ambient PM_2.5_ concentrations averaged over the 48 hr before the examination were associated with a reduction HF, with negative, albeit nonsignificant, associations were seen with SDNN and LF (Park et al. 2005). In the present work, based on longer follow-up and additional HRV measurements, we were also able to show a significant effect on SDNN, as well as a more pronounced, although still nonsignificant, negative association with LF.

As part of our examination of oxidative stress we have previously shown that particles had no effect on HRV in subjects with the functional *GSTM1* polymorphism (*GSTM1*–wild-type) but had a substantially increased effect in those with the deletion (*GSTM1*-null) (Schwartz et al. 2005b). Similarly, we showed that statin use and obesity, which both modify ROS production, altered PM_2.5_ effects on HRV (Schwartz et al. 2005b), thus confirming the critical role of oxidative stress pathways. In this article, we extend those results by showing a three-way interaction with genetic modifiers of response to oxidative stress.

Although particle exposure has also been linked with activation of inflammatory pathways (Baccarelli et al. 2007b; Liao et al. 2004; Peters et al. 2001), alterations in blood coagulation (Baccarelli et al. 2007a; Liao et al. 2005), endothelial injury and dysfunction (Brook et al. 2002; Ikeda et al. 1998), and alterations in the autonomic control of the heart (Creason et al. 2001; Gold et al. 2000; Liao et al. 2004a), our findings suggest that genetic variations in oxidative stress pathways play a critical role in the cardiovascular effects of airborne particles.

Rodents exposed to concentrated urban particles evinced increased reactive oxygen species in both the lung and the heart (Gurgueira et al. 2002), an effect muted by preadministration of *N*-acetylcysteine, a glutathione precursor and potent antioxidant (Rhoden et al. 2004). Inhalation of particles produces oxidative stress directly or via acute pulmonary inflammation, thus causing a series of events, such as the production of proinflammatory mediators, an increase of extracellular calcium influx, and the disruption of nitric oxide regulation (Stone et al. 2000; Thomas et al. 2001), that may impair autonomic function and hence HRV. Diesel particles have also been shown to increase oxidative stress in endothelial tissue, inducing the production of HMOX-1 (Furuyama et al. 2006). The viability of cell cultures of microvascular endothelial cells was impaired by diesel particles with an accompanying large increase in induction of HMOX-1 (Hirano et al. 2003); this process was blunted by *N*-acetylcysteine. Woodsmoke particles have also been shown to deplete intracellular glutathione and upregulate HMOX-1 activity in endothelial cells (Liu et al. 2005). Our results showing interactions of particles with *GSTM1* deletion and microsatellite (GT)n repeat length in the gene coding for HMOX-1 are consistent with these laboratory findings that suggest a prominent role of ROS in particle toxicity.

HMOX-1, the inducible heme oxygenase isoform, is expressed in multiple tissues, including vascular smooth muscle and endothelial cells (Exner et al. 2004). *HMOX-1* expression has been shown to be upregulated in rat heart microvessel endothelial cells exposed to organic extracts of diesel exhaust particles (Furuyama et al. 2006), an effect that is likely to represent a response directed against ROS production (Morita 2005). Large individual differences in the ability to modulate the quantitative level of HMOX-1 activity in response to a given stimulus have been described, which correlate with differences in the length of a microsatellite (GT)n repeat in the 5′ flanking region of the *HMOX-1* gene (Exner et al. 2004; Hirai et al. 2003; Yamada et al. 2000). The purine–pyrimidine alternating sequence in the (GT)n repeat has the potential to assume Z-DNA conformation, a left-handed double-helix structure that is thermodynamically unfavorable compared with B-DNA conformation (Rich et al. 1984) and has been described as negatively affecting transcriptional activity (Delic et al. 1991; Naylor and Clark 1990). Yamada and co-workers demonstrated by transient-transfection assay in cultured cell lines that the larger the number of (GT)n repeats in the *HMOX-1* gene promoter, the lower is the *HMOX-1* inducibility by ROS (Yamada et al. 2000).

In our study, *HMOX-1* microsatellite (GT)n repeat length appeared to modulate the effects of PM_2.5_ on autonomic function, as measured by HRV variability. PM_2.5_ exhibited a negative correlation with SDNN, HF, and LF in individuals with ≥ 25 repeats, whereas no effect was seen in subjects with < 25 repeats. Furthermore, the strongest effects of PM_2.5_ were found in subjects that were lacking efficiency in antioxidant responses due to the combination of the *GSTM1* deletion and ≥ 25 microsatellite (GT)n repeats in the *HMOX-1* promoter. While the *p*-values for statistical interaction between PM_2.5_ and *GSTM1* and between PM_2.5_ and *HMOX-1* were not significant, the tests for the three-way interaction among PM_2.5_, *GSTM1*, and *HMOX-1* were highly significant, confirming that the stronger effect modification of PM_2.5_ effects on HRV is seen when both genes are considered. The statistical modeling we used to fit the three-way interaction tested whether the size of PM_2.5_ effects in carriers of *GSTM1* null and at least one long *HMOX-1* repeat was 2 times larger than that observed in subjects carrying either *GSTM1* null or one long *HMOX-1* repeat. Thus, our data strongly suggest that particle exposure interacts with individual variations in the antioxidant response pathway to determine its effects on HRV.

A potential limitation of this study is that we used ambient PM_2.5_ concentrations from a single monitoring site as a surrogate for recent exposure to PM_2.5_. A recent study comparing ambient concentrations at this site with personal exposures in Boston has shown a high longitudinal correlation (Sarnat et al. 2005) between the two measurements; the study also reported that PM_2.5_ concentrations were spatially homogeneous over the Boston area. This suggests that our use of ambient concentrations is reasonable and the resulting exposure error is likely to be nondifferential. In our analyses, we considered several potential confounding factors that may have influenced HRV measures, as we adjusted our models for age, BMI, mean arterial pressure, fasting blood glucose, cigarette smoking, alcohol consumption, room temperature, outdoor apparent temperature, season, and use of beta-blockers, calcium channel blockers, and ACE inhibitors. Therefore, chances that the observed associations reflected bias due to confounders are minimized.

Our results can be generalized only to an aged population that consists of older males who are almost all white. The effect on women and children as well as different ethnic groups should be addressed in future studies, particularly in relation to the exposure of different population groups to PM_2.5_ with varying geographic location, occupation, socioeconomic status, and behavioral characteristics. Other health outcomes including respiratory responses may also be affected by responses to ROS in an interaction with PM_2.5_ exposure. Our findings provide new information to guide research on the breadth of the effect of PM_2.5_ exposure.

## Figures and Tables

**Table 1:** Anthropometric, clinical characteristics, and heart rate variability parameters [mean ± SD or *n* (%)] of the study population, by *GSTM1* polymorphism status and *HMOX-1* microsatellite repeat length.

			By *GSTM1* and *HMOX-1* polymorphisms (*n* = 476)			
Variable	All subjects (*n* = 539)	All subjects analyzed for both *HMOX-1* and *GSTM1* (*n* = 476)	*GSTM1* wt *HMOX-1* < 25 repeats^a^ (*n* = 20)	*GSTM1* null *HMOX-1* < 25 repeats^a^ (*n* = 24)	*GSTM1* wt *HMOX-1* ≥ 25 repeats^b^ (*n* = 204)	*GSTM1* null *HMOX-1* ≥ 25 repeats^b^ (*n* = 228)
Age (years)	72.8 ± 6.6	73.0 ± 6.7	72.5 ± 4.8	73. 2 ± 5.9	73.0 ± 6.8	73.1 ± 6.82
BMI (kg/m^2^)	28.2 ± 4.1	28.0 ± 4.1	27.8 ± 4.7	28.3 ± 3.3	28.1 ± 4.4	28.0 ± 3.90
Systolic blood pressure (mm Hg)	130.6 ± 16.3	130.5 ± 16.7	131.4 ± 16.1	133.8 ± 16.3	129.5 ± 15.7	131.0 ± 17.8
Diastolic blood pressure (mm Hg)	74.9 ± 9.7	74.7 ± 9.7	74.3 ± 8.5	76.3 ± 7.1	74.7 ± 9.7	74.7 ± 10.0
Mean arterial pressure (mm Hg)	93.5 ± 10.6	93.3 ± 10.7	93.3 ± 9.5	95.5 ± 8.6	92.9 ± 10.5	93.4 ± 11.2
Heart rate (beats/min)	70.7 ± 6.8	71.0 ± 6.8	72.0 ± 4.6	70.6 ± 6.5	70.5 ± 6.8	71.2 ± 7.1
Fasting blood glucose (mg/dL)	108.4 ± 28.4	109.2 ± 29.5	108.2 ± 35.8	115.2 ± 32.7	112.2 ± 35.4	105.9 ± 21.5
Total cholesterol (mg/dL)	194.9 ± 36.7	194.8 ± 37.3	187.3 ± 32.9	202.3 ± 37.8	196.7 ± 35.3	192.9 ± 39.3
HDL (mg/dL)	49.5 ± 13.3	49.8 ± 13.3	52.2 ± 15.2	49.4 ± 13.1	49.3 ± 12.5	49.9 ± 13.8
Triglyceride (mg/dL)	131.8 ± 72.2	130.5 ± 72.8	118.4 ± 66.3	125.9 ± 60.2	130.9 ± 67.5	131.8 ± 79.1
Smoking status [*n* (%)]						
Never smoker	165 (30.6)	145 (30.5)	6 (30.0)	5 (20.8)	71 (34.8)	63 (27.6)
Current smoker	27 (5.0)	23 (4.8)	3 (15.0)	2 (8.3)	6 (2.9)	12 (5.3)
Former smoker	347 (64.4)	308 (64.7)	11 (55.0)	17 (70.8)	127 (62.3)	153 (67.1)
Alcohol intake (≥ 2 drinks/day) [*n* (%)]	102 (18.9)	89 (18.7)	2 (10.0)	4 (16.7)	42 (20.6)	41 (18.0)
Diabetes mellitus, *n* (%)	80 (14.8)	75 (15.8)	5 (25.0)	6 (25.0)	38 (18.6)	26 (11.4)
CHD history [*n* (%)]	153 (28.4)	138 (29.0)	5 (25.0)	6 (25.0)	58 (28.4)	69 (30.3)
Stroke history [*n* (%)]	33 (6.1)	29 (6.1)	1 (5.0)	1 (4.2)	17 (8.3)	10 (4.4)
Hypertension [*n* (%)]	377 (69.9)	331 (69.5)	14 (70.0)	15 (62.5)	139 (68.1)	163 (71.5)
Use of beta-blocker [*n* (%)]	181 (33.6)	157 (33.0)	7 (35.0)	5 (20.8)	66 (32.4)	79 (34.7)
Use of Ca channel blocker [*n* (%)]	70 (13.0)	61 (12.8)	1 (5.0)	2 (8.3)	30 (14.7)	28 (12.3)
Use of ACE inhibitor [*n* (%)]	112 (20.8)	105 (22.1)	5 (25.0)	2 (8.3)	37 (18.1)	61 (26.8)
Heart rate variability^c^						
Log_10_ SDNN (msec)	1.52 ± 0.25	1.52 ± 0.25	1.53 ± 0.24	1.48 ± 0.21	1.52 ± 0.26	1.53 ± 0.25
Log_10_ HF (msec^2^)	1.90 ± 0.65	1.90 ± 0.65	1.84 ± 0.52	1.74 ± 0.58	1.92 ± 0.65	1.92 ± 0.67
Log_10_ LF (msec^2^)	2.00 ± 0.53	2.00 ± 0.53	1.99 ± 0.48	1.93 ± 0.48	2.00 ± 0.56	2.01 ± 0.52
Environmental variables						
PM_2.5_^d^ (μg/m^3^)	11.7 ± 7.8	11.6 ± 7.9	11.9 ± 5.8	12.3 ± 12.2	11.6 ± 7.8	11.5 ± 7.7
Apparent temperature^d^ (°C)	11.1 ± 10.0	10.9 ± 10.0	12.4 ± 9.9	12.6 ± 9.8	10.7 ± 10.2	10.7 ± 9.8
Room temperature (°C)	24.3 ± 1.8	24.3 ± 1.8	25.0 ± 0.9	24.8 ± 1.8	24.3 ± 1.7	24.2 ± 1.9

Abbreviations: LDL, low-density lipoprotein.

a Carriers of < 25 microsatellite (GT) repeats in both alleles.

b Carriers of ≥ 25 microsatellite (GT) repeats in at least one allele.

c Standard deviation of SDNN, power in HF (0.15–0.4 Hz) and LF (0.04–0.15 Hz) computed using a fast Fourier transform algorithm.

d Average of hourly measurements of PM_2.5_ and apparent temperature during the 48 hr before the HRV measurement.

**Table 2:** Adjusted percent change (95% CI) of heart rate variability (HRV) for each 10 μg/m^3^ of PM_2.5_ in the 48 hr before the measurement, by *HMOX-1* micro-satellite repeat length or *GSTM1* polymorphism.

		Model 2 PM_2.5_ effect by *GSTM1*		Model 3 PM_2.5_ effect by *HMOX-1* microsatellite length	
HRV measurement^a^	Model 1Main effect of PM_2.5_	*GSTM1* wild-type	*GSTM1* null	*HMOX-1* < 25 repeats^b^	*HMOX-1* ≥ 25 repeats^c^
log_10_ SDNN	−6.8%	−2.0%	−10.5%	7.4%	−8.5%
	(−12.9 to −0.2)	(−11.3 to 8.3)	(−18.2 to −2.2)	(−8.7 to 26.2)	(−14.8 to −1.8)
	*p* = 0.0436	*p* = 0.6908	*p* = 0.0150	*p* = 0.3891	*p* = 0.0137
		*p-*interaction = 0.1382		*p-*interaction = 0.0594	
log_10_ HF	−17.3%	−4.0%	−24.2%	8.9%	−20.1%
	(−30.0 to −2.3)	(−24.8 to 22.6)	(−39.2 to −5.5)	(−27.1 to 62.8)	(−32.9 to −5.0)
	*p* = 0.0263	*p* = 0.7442	*p* = 0.0139	*p* = 0.6759	*p* = 0.0115
		*p-*interaction = 0.1178		*p-*interaction = 0.1408	
log_10_ LF	−11.2%	−0.6%	−17.0%	14.0%	−14.0%
	(−22.8 to 2.2)	(−19.0 to 22.0)	(−31.0 to −0.2)	(−18.6 to 59.5)	(−25.7 to −0.5)
	*p* = 0.0986	*p* = 0.9545	*p* = 0.0478	*p* = 0.4465	*p* = 0.0430
		*p-*interaction = 0.1536		*p-*interaction = 0.1102	

All models adjusted for age, BMI, mean arterial pressure, fasting blood glucose, cigarette smoking (never/former/current), alcohol consumption (two or more drinks a day, yes/no), use of beta-blockers, use of calcium channel blockers, use of ACE inhibitor, room temperature, season, and 48-hr moving average of outdoor apparent temperature.

a Standard deviation of SDNN, power in HF (0.15–0.4 Hz) and LF (0.04–0.15 Hz) computed using a fast Fourier transform algorithm.

b Carriers of < 25 microsatellite (GT) repeats in both alleles.

c Carriers of ≥ 25 microsatellite (GT) repeats in at least one allele.

**Table 3:** Effect modification of *HMOX-1* microsatellite repeat length and *GSTM1* combinations on the adjusted percent change (95% CI) of HRV for each 10 μg/m^3^ of PM_2.5_ in the 48 hr before the measurement.

HRV measurement^a^	*HMOX-1* < 25 repeats^b^*GSTM1* wild-type (no. of visits = 26^d^)	*HMOX-1* < 25 repeats^b^ and *GSTM1* null or *HMOX-1* ≥ 25 repeats^c^ and *GSTM1* wild-type (no. of visits = 309^d^)	*HMOX-1* ≥ 25 repeats^c^*GSTM1* null (no. of visits = 303^d^)	Test for interaction
Log_10_ SDNN	28.7% (−12.0 to 88.2) *p* = 0.1937	−2.7% (−11.5 to 6.8) *p* = 0.5620	−12.7% (−20.6 to −3.9) *p* = 0.0059	*p* = 0.0084
Log_10_ HF	45.6% (−43.5 to 275.3) *p* = 0.4370	−5.8% (−25.3 to 18.8) *p* = 0.6153	−27.8% (−43.0 to −8.5) *p* = 0.0073	*p* = 0.0108
Log_10_ LF	68.5% (−23.3 to 270.4) *p* = 0.1944	−2.8% (−19.9 to 18.0) *p* = 0.7722	−20.1% (−34.5 to −2.7) *p* = 0.0261	*p* = 0.0385

All models adjusted for age, BMI, mean arterial pressure, fasting blood glucose, cigarette smoking (never/former/current), alcohol consumption (two or more drinks a day, yes/no), use of beta-blockers, use of calcium channel blockers, use of ACE inhibitor, room temperature, season, and 48-hr moving average of outdoor apparent temperature.

a Standard deviation of SDNN, power in HF (0.15–0.4 Hz) and LF (0.04–0.15 Hz) computed using a fast Fourier transform algorithm.

b Carriers of < 25 microsatellite (GT) repeats in both alleles.

c Carriers of ≥ 25 microsatellite (GT) repeats in at least one allele.

d Number of HRV measurements.
